# More Realistic Forecasting of Future Life Events After Psilocybin for Treatment-Resistant Depression

**DOI:** 10.3389/fpsyg.2018.01721

**Published:** 2018-10-12

**Authors:** Taylor Lyons, Robin Lester Carhart-Harris

**Affiliations:** Psychedelic Research Group, Department of Medicine, Imperial College London, London, United Kingdom

**Keywords:** treatment-resistant depression, psilocybin, cognitive bias, pessimism, forecasting

## Abstract

**Background:** Evidence suggests that classical psychedelics can promote enduring changes in personality, attitudes and optimism, as well as improvements in mental health outcomes.

**Aim:** To investigate the effects of a composite intervention, involving psilocybin, on pessimism biases in patients with treatment-resistant depression (TRD).

**Methods:** Patients with TRD (*n* = 15) and matched, untreated non-depressed controls (*n* = 15) performed the Prediction Of Future Life Events (POFLE) task. The POFLE task requires participants to predict the likelihood of certain life events occurring within a 30-day period, after which the actual rate of event occurrence is reported; this gives an index of potential pessimism versus optimism bias. Psilocybin was administered in two oral dosing sessions (10 and 25 mg) one week apart. Main outcome measures were collected at baseline and one week after the second dosing session.

**Results:** Patients showed a significant pessimism bias at baseline [*t*(14) = -3.260, *p* = 0.006; 95% CI (-0.16, -0.03), *g* = 1.1] which was related to the severity of their depressive symptoms (*r_s_* = -0.55, *p* = 0.017). One week after psilocybin treatment, this bias was significantly decreased [*t*(14) = -2.714, *p* = 0.017; 95% CI (-0.21, -0.02), *g* = 0.7] and depressive symptoms were greatly improved [*t*(14) = 7.900, *p* < 0.001; 95% CI (16.17, 28.23), *g* = 1.9]; moreover, the magnitude of change in both variables was significantly correlated (*r* = -0.57, *p* = 0.014). Importantly, post treatment, patients became significantly more accurate at predicting the occurrence of future life events [*t*(14) = 1.857, *p* = 0.042; 95% CI (-0.01, 0.12), *g* = 0.6] whereas no such change was observed in the control subjects.

**Conclusion:** These findings suggest that psilocybin with psychological support might correct pessimism biases in TRD, enabling a more positive and accurate outlook.

## Introduction

Major depressive disorder (MDD) is one of the foremost contributors to the overall global burden of disease (Marcus et al., 2012). Approximately 30% of MDD patients suffer from treatment-resistant depression (TRD) (Gaynes, 2009; Al-Harbi, 2012) and much of the burden associated with MDD can be accounted for by treatment resistance (Greden, 2001). Cognitive therapy is the most widely studied and practiced psychotherapeutic intervention for MDD (Butler et al., 2006). The cognitive-bias model of depression states that patients have an unrealistic negative perspective of themselves and the world more generally (Beck, 1967, 1976; Beck and Rush, 1979). Somewhat in contrast to this, the depressive realism hypothesis states that depressed patients actually see themselves and the world in a more realistic way than the general population – who, the hypothesis maintains, are somewhat inaccurate and unrealistic in their optimism (Alloy and Abramson, 1979). The cognitive triad—which comprises a negative attitude towards oneself, the environment and the future (Beck, 1967)—partly forms the theory upon which cognitive therapy for MDD is based. A key cognitive feature in MDD is that patients expect the future to be bleak and anticipate unfavorable outcomes when facing life events of unknown emotional impact. Early research has shown that MDD patients have more dysfunctional attitudes, feelings of hopelessness, negative thoughts and pessimism than healthy individuals (Peterson and Seligman, 1984; Hollon et al., 1986; Beck et al., 1988; Hill et al., 1989). More recent findings have since supported the cognitive-bias model of depression over the depressive realism hypothesis, demonstrating, for example, that MDD patients exhibit pessimistic biases when anticipating future life events (Strunk et al., 2006; Strunk and Adler, 2009).

As MDD is associated with excessive pessimism, it is interesting to note that psilocybin—a naturally occurring psychedelic compound derived from *Psilocybe* mushrooms—has been shown to increase optimism, psychological wellbeing, trait openness, and life satisfaction in an enduring way following just a single dose in healthy populations (Griffiths et al., 2008; MacLean et al., 2011). Increased optimism and wellbeing have also been found for weeks to months after a controlled administration of LSD to healthy volunteers (Carhart-Harris et al., 2016b; Liechti, 2017). Functional brain imaging of the psychedelic state has implicated brain regions linked to MDD (Carhart-Harris et al., 2012), including the subgenual prefrontal cortex (sgPFC) and default mode network (DMN) (Hamilton et al., 2015). Moreover, the key receptor associated with the action of psychedelics, the serotonin 2A receptor [5-HT_2A_R (Glennon et al., 1984), has its highest expression in DMN regions (Beliveau et al., 2017) – and 5-HT_2A_R binding has been reliably linked with trait pessimism (Meyer et al., 2003; Bhagwagar et al., 2006) and neuroticism (Frokjaer et al., 2008, 2010)]. It has been hypothesized that deficient signaling at the 5-HT_2A_R may confer an inflexible and pessimistic mind-set, and that increased 5-HT_2A_R signaling may serve to “lubricate the mind,” relaxing prior beliefs to aid new learning (Carhart-Harris and Nutt, 2017); see Matias et al. (2017) for more empirical support.

Recent years have witnessed a resurgence of interest in the ability of classical psychedelic compounds to treat a range of disorders, including MDD (Dos Santos et al., 2016; Carhart-Harris and Goodwin, 2017). Randomized controlled trials (RCTs) have demonstrated that psilocybin can rapidly alleviate depression and anxiety in patients with life-threatening cancer, with sustained effects for 6 months (Griffiths et al., 2016; Ross et al., 2016). A recent feasibility study of ours found that psilocybin with psychological support was well tolerated, and associated with rapid and marked reductions in depressive symptoms in TRD patients (Carhart-Harris et al., 2016a, 2017a), thus endorsing it’s potential as a safe and effective treatment for such clinical populations, albeit with important caveats (Carhart-Harris et al., 2018).

The present study sought to investigate the presence of pessimism in TRD patients, in line with the depressive-bias hypothesis, and the ability of psilocybin to alleviate this. It was hypothesized that the TRD patients would indeed show a significant pessimism bias that could be effectively treated with psilocybin therapy. Relevant measures of pessimism were given at baseline and one-week post-dosing. To demonstrate robustness to order confounds, TRD patients’ data were compared with those from non-treated healthy control subjects matched in terms of age, gender and education tested over an equivalent time period.

## Materials and Methods

### Ethical Approvals

This study received a favorable opinion from NRES London-West London, was sponsored by Imperial College London, and was carried out in accordance with Good Clinical Practice Guidelines. The National Institute for Health Research/Wellcome Trust ICRF provided site-specific approval and the Medicines and Healthcare products Regulatory Agency reviewed and approved this research. All patients provided written informed consent.

### Study Design and Participants

This open-label pilot study with a mixed-model design investigated the effects of psilocybin on measures of depressive symptoms and cognitive biases in TRD patients versus healthy non-treated control subjects matched in terms of age, gender and education tested over an equivalent time period. The patients and research team were not masked to treatment assignment. All TRD patients (*n* = 15) were administered psilocybin in two dosing sessions: an initial safety dose (10 mg) and a subsequent treatment dose (25 mg) 1 week later. Please refer to Carhart-Harris et al. (2016a) for a full description of the procedures and dosing sessions. Physically and mentally healthy control subjects (*n* = 15) were recruited via word of mouth and were not administered psilocybin.

### Drug

Psilocybin was obtained from THC Pharm GmbH (Frankfurt, Germany) and formulated (5 mg psilocybin per size 0 capsule) by Guy’s and St Thomas’ Hospitals’ Pharmacy Manufacturing Unit (London, United Kingdom). Home Office approvals for storing and dispensing Schedule One drugs were obtained.

### Outcome Measures

**FIGURE 1:**
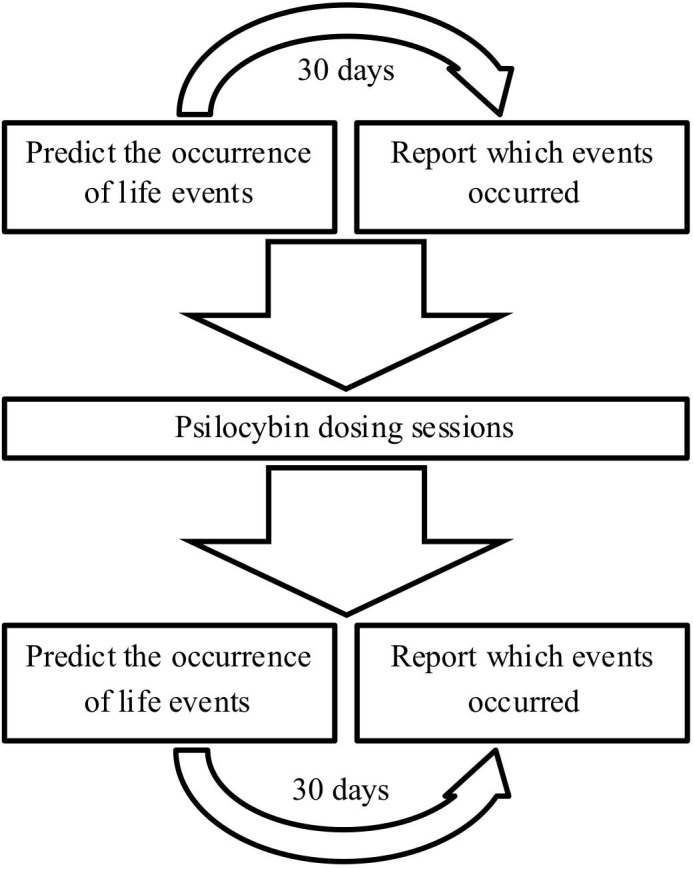
Schematic diagram of the study design.

Depressive symptoms were measured in all study participants using the Beck Depression Inventory (BDI)—a self-report rating inventory with 21 items that measures characteristic attitudes and symptoms of depression (Beck et al., 1961). Cognitive biases were measured using the Prediction Of Future Life Events task (POFLE; Strunk et al. (2006)). The POFLE involves 40 different life events, 20 desirable and 20 undesirable, and patients must predict the probability of each occurring within the next 30 days [see Strunk et al. (2006) for the scoring procedures]. The POFLE was split into two versions, A and B, each containing 20 items. All patients received both versions in balanced order; half of the patients received POFLE version A at screening and POFLE version B at the follow-up, and the other half vice versa. Patients were contacted 30 days after completing each version to determine which of the events actually occurred. All of the above outcome measures were assessed at screening and then again at the follow-up (**Figure 1**).

### Statistical Analysis

All statistical analyses were performed using SPSS version 23.0 (IBM Corp., Armonk, NY, United States). Within-group comparisons were performed using two-tailed paired *t*-tests for parametric data and Wilcoxon signed ranks tests for non-parametric data. Between-group comparisons were performed using two-tailed independent *t*-tests for parametric data and Mann-Whitney *U* tests for non-parametric data. Depressive symptoms over time were analyzed using the non-parametric Friedman Test with Dunn’s correction for multiple *post hoc* comparisons. Correlations were analyzed using Pearson’s correlation coefficient for parametric data and Spearman’s rho for non-parametric data. We provide 95% CIs around the mean differences. Effect sizes were calculated using the Hedges’ *g* formula.

## Results

### Demographics

Of the total 30 participants who took part in this study, the majority were Caucasian (93.33%) and men (73.33%) with a post-secondary level of education (80%). Self-reported depressive symptoms were collected using the Beck Depression Inventory (BDI) for all study participants. Of the 15 clinically assessed patients with TRD, the Hamilton Depression Scale (HAM-D) scores ranged from 18 to 29 (*M* = 23.73, *SD* = 4.70) and the Montgomery–Åsberg Depression Rating Scale scores from 24 to 42 (*M* = 32.33, *SD* = 5.14); thus, all patients recruited were diagnosed with TRD of at least moderate severity, with most (*n* = 10) meeting criteria for severe depression (HAM-D > 24; BDI > 30). Control subjects were not clinically assessed because they were deemed physically and mentally healthy. A breakdown of the participant demographic details and depressive scores at baseline can be found in **Table 1**.

**Table 1:** Study participant demographics and baseline depression scores.

Characteristic	Control subjects (*n* = 15)	TRD patients (*n* = 15)	All participants (*N* = 30)	*P* value
Gender (% male)	60.0%	73.3%	66.7%	0.439^a^
Age in years (mean, SEM)	37.6 (3.7)	45.4 (2.9)	41.5 (2.4)	0.109^b^
Ethnicity (%)				0.309^a^
White	100.0%	93.3%	96.7%	
Black	0%	6.7%	3.3%	
Education (%)				0.497^a^
Secondary School	6.7%	20.0%	13.3%	
Undergraduate	53.3%	53.3%	53.3%	
Postgraduate	40.0%	26.7%	33.3%	
Baseline scores (mean, SEM)				
BDI	4.07 (1.10)	34.33 (1.92)	NA	<0.001^c^


*^a^ Chi-square test. ^b^ t test. ^c^ U test.*

### Depressive Symptoms

At baseline, patients (*M* = 34.33, *SD* = 7.44) scored significantly higher than controls (*M* = 3.67, *SD* = 3.83) on the BDI (*U* = 0.0E0, *p* < 0.001, *g* = 5.0). The patients showed a significant decrease in BDI scores one week after the psilocybin sessions (*M* = 12.13, *SD* = 9.80) compared to baseline [*M* = 34.33, *SD* = 7.44; *t*(14) = 7.900, *p* < 0.001; 95% CI (16.17, 28.23), *g* = 1.9]. There was no significant difference found in the controls’ baseline (*M* = 3.67, *SD* = 3.83) versus follow-up BDI scores (*M* = 2.73, *SD* = 3.41; *Z* = -1.071, *p* = 0.284). These results confirm that depressive symptoms were significantly reduced for the patients 1 week after psilocybin treatment, but did not change over an equivalent period for the non-treated control subjects.

**FIGURE 2:**
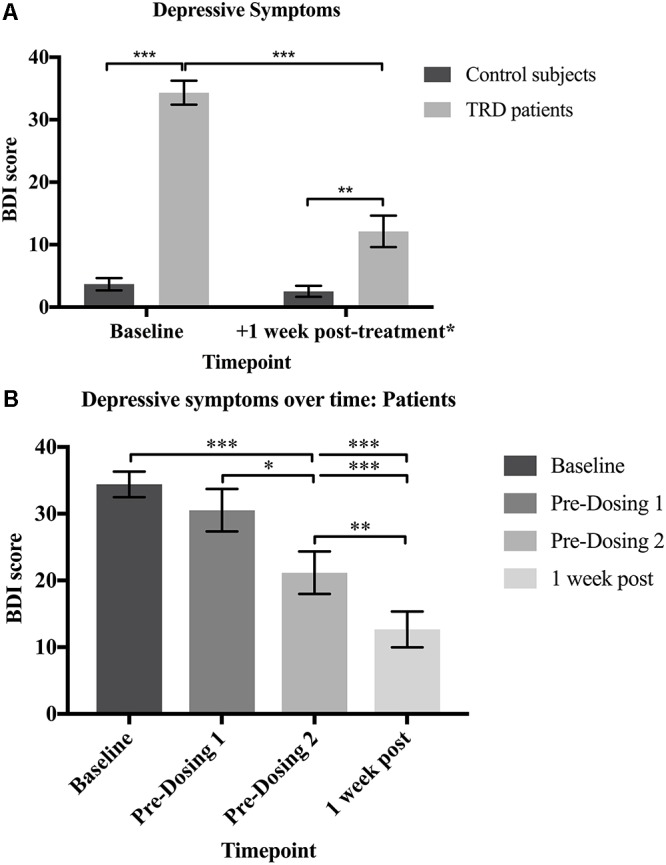
**(A)** Depressive symptoms. Patients had significantly higher BDI scores than controls at baseline (*U* = 0.0E0, *p <* 0.001; *g* = 5.0). The patients’ BDI scores were significantly reduced 1 week after psilocybin treatment [*t*(14) = 7.900, *p* < 0.001; 95% CI (16.17, 28.23), *g* = 1.9], but remained significantly greater than the controls (*U* = 34.50, *p* = 0.001; *g* = 1.1). **(B)** Depressive symptoms over time: Patients. There was a significant reduction in the patients’ BDI scores over time [*X^2^*(3, *N* = 15) = 32.35, *p* < 0.0001]. Baseline BDI scores did not significantly differ from scores at the beginning of dosing day 1 (*p* = 0.944), confirming that the depressive symptoms had not naturally declined prior to psilocybin treatment. BDI scores were significantly reduced on dosing day 2 when compared with baseline (*p* < 0.001) and dosing day 1 (*p* = 0.033) scores. One-week after both dosing sessions, BDI scores were significantly reduced when compared to baseline (*p* < 0.001), dosing day 1 (*p* < 0.001) and dosing day 2 (*p* = 0.009) scores. Data expressed as mean ± SEM (*p* < 0.05^∗^; *p* < 0.01^∗∗^; *p* < 0.001^∗∗∗^). Note: Time (days) between: (i) baseline and dosing day 1 (*M* = 35.0, *SD* = 23.3); (ii) dosing day 1 and dosing day 2 (*M* = 7.9, *SD* = 2.6) (iii) dosing day 2 and one-week post follow-up (*M* = 7.8, *SD* = 2.5; (iv) baseline and one-week post follow-up (*M* = 50.8; *SD* = 25.3).

Despite the significant improvement, patients’ post-treatment BDI scores (*M* = 12.13, *SD* = 9.80) continued to significantly differ from the controls’ follow-up values (*M* = 4.29, *SD* = 4.35; *U* = 34.50, *p* = 0.001, *g* = 1.1). These results suggest that the patients’ depressive symptoms were largely reduced but did not quite reach levels comparable with healthy controls one week after psilocybin treatment (**Figure 2A**).

The patients’ depressive symptoms were also measured prior to the administration of psilocybin at the beginning of dosing days 1 and 2. There was a significant reduction in the patients’ BDI scores over time [*X^2^*(3, *N* = 15) = 32.35, *p* < 0.0001]. *Post hoc* comparisons with Dunn’s correction showed no significant difference in baseline (*M* = 34.33, *SD* = 7.44) versus dosing day 1 (*M* = 30.53, *SD* = 12.36; *p* = 0.944) BDI scores. This confirms that depressive symptoms did not naturally decline from baseline prior to psilocybin treatment. However, BDI scores were significantly reduced on dosing day 2 (*M* = 21.13, *SD* = 12.93) when compared with baseline (*M* = 34.33, *SD* = 7.44; *p* < 0.001) and dosing day 1 (*M* = 30.53, *SD* = 12.36; *p* = 0.033) scores. This demonstrates a reduction in the patients’ depressive symptoms 1 week after receiving the first dose of psilocybin. One-week after both dosing sessions (*M* = 12.67, *SD* = 10.40), BDI scores were significantly reduced when compared to baseline (*M* = 34.33, *SD* = 7.44; *p* < 0.001), dosing day 1 (*M* = 30.53, *SD* = 12.36; *p* < 0.001) and dosing day 2 (*M* = 21.13, *SD* = 12.93; *p* = 0.009) scores. This confirms that the patients’ depressive symptoms were reduced even further following each dose of psilocybin (**Figure 2B**).

### Predicting Future Life-Events Before Psilocybin Treatment

Before proceeding to testing the specific hypotheses of this study, some description of the prediction and event occurrence data that is required for bias computation is warranted (see **Supplementary Material**). As the POFLE contains both desirable and undesirable life events, it is most informative to consider the data split by the desirability of these life events. When predicting life events at baseline, the patients gave similar probability estimates for desirable (*M* = 0.29, *SD* = 0.15) and undesirable life events [*M* = 0.23, *SD* = 0.15, *t*(14) = 1.037, *p* = 0.317; 95% CI (-0.07, 0.19)]. Contrary to their predictions however, patients reported experiencing more desirable (*M* = 4.60, *SD* = 1.76) than undesirable events [*M* = 1.40, *SD* = 1.35, *t*(14) = 5.967, *p* < 0.001; 95% CI (2.05, 4.35), *g* = 1.5] within the ensuing 30-day period after their baseline predictions. This demonstrates that the patients predicted a negative future that was not borne out by their experience.

Control subjects gave higher probability estimates for desirable (*M* = 0.57, *SD* = 0.09) than undesirable life events [*M* = 0.22, *SD* = 0.16; *t*(14) = 8.492, *p* < 0.001; 95% CI (0.26, 0.43), *g* = 2.0] at baseline and in line with their predictions, reported the occurrence of more desirable (*M* = 5.60, *SD* = 1.45) than undesirable [*M* = 2.33, *SD* = 1.84; *t*(14) = 4.859, *p* < 0.001; 95% CI (1.8, 4.7), *g* = 1.2] events within the ensuing 30 days. This suggests that the controls could realistically forecast the occurrence of future life events – most of which were desirable.

Between-group comparisons revealed that patients predicted significantly less desirable events than control subjects at baseline [*M* = 0.29, *SD* = 0.15 vs *M* = 0.57, *SD* = 0.09; *t*(28) = 5.820, *p* < 0.001; 95% CI (0.17, 0.36), *g* = 2.2], but there was no significant difference found between patients (*M* = 0.23, *SD* = 0.15) and controls (*M* = 0.22, *SD* = 0.16) in their predictions of undesirable events (*U* = 108.000, *p* = 0.852). Therefore, despite the occurrence of relatively more desirable than undesirable life events occurring within the ensuing 30-day period, the patients (wrongly) expected an equal number of desirable and undesirable life events, and predicted significantly less desirable life events than the control subjects. Both results are consistent with an unrealistic pessimism bias in the patients prior to receipt of psilocybin, in which they were especially prone to underestimate the (true) likelihood of desirable future life events.

There were no significant between-groups differences found in the rates at which desirable (patients: *M* = 4.60, *SD* = 1.76; controls: *M* = 5.60, *SD* = 1.45; *U* = 75.000, *p* = 0.113) and undesirable (patients: *M* = 1.40, *SD* = 1.35 controls: *M* = 2.33, *SD* = 1.84; *U* = 78.500, *p* = 0.148) events occurred within the 30-day period from baseline. These findings reflect that both groups were experiencing similar life circumstances, despite patients predicting a more negative reality.

### Predicting Future Life-Events After Psilocybin-Treatment (Second 30-Day Period)

In contrast to their pre-treatment pessimism, post-treatment, patients gave significantly higher probability estimates for desirable (*M* = 0.44, *SD* = 0.20) than undesirable life events [*M* = 0.25, *SD* = 0.22, *t*(14) = 2.322, *p* = 0.036; 95% CI (0.01, 0.35), *g* = 0.6]. Justifying this difference, the patients also reported a higher percentage of desirable events (*M* = 3.80, *SD* = 2.57) actually occurring within this (second and post-treatment) 30-day period – relative to undesirable events [*M* = 1.40, *SD* = 1.18; *t*(14) = 3.485, *p* = 0.004; 95% CI (0.92, 3.88), *g* = 1.3]; suggestive of an improved accuracy in predicting future life events (see below for more specific accuracy measures).

As they did at baseline, control subjects gave higher probability estimates for desirable (*M* = 0.57, *SD* = 0.14) than undesirable (*M* = 0.20, *SD* = 0.12) life events at the follow-up [*t*(14) = 8.399, *p* < 0.001; 95% CI (0.28, 0.46), *g* = 2.0], and did not differ from baseline in their second predictions of desirable [*t*(14) = -0.0001, *p* = 1.000; 95% CI (-0.07, 0.07)] or undesirable events [*t*(14) = 0.686 *p* = 0.504; 95% CI (-0.06, 0.12)]. Consistent with these predictions, control subjects reported the occurrence of more desirable (*M* = 5.53, *SD* = 1.19) than undesirable (*M* = 1.80, *SD* = 1.78) events within the ensuing 30 days from follow-up [*t*(14) = 8.071, *p* < 0.001; 95% CI (2.74, 4.73), *g* = 2.0].

Contrary to what was seen at baseline, there were no significant between-groups differences in the predictions of both desirable [*t*(28) = 1.972, *p* = 0.059; 95% CI (-0.01, 0.25)] and undesirable events (*U* = 95.000, *p* = 0.468) for the second block of predictions. Further, there were no significant between-groups differences found in the rates at which desirable (*U* = 70.000, *p* = 0.072) and undesirable (*U* = 105.000, *p* = 0.741) events actually occurred within the second 30-day period.

In summary, after treatment with psilocybin, consistent with what was actually reported to have happened during the ensuing 30-day period, TRD patients (rightly) expected more desirable than undesirable life events to occur – and thus began to make more accurate predictions of future life events – in line with the behavior of healthy control subjects.

### Accuracy of Forecasts

**FIGURE 3:**
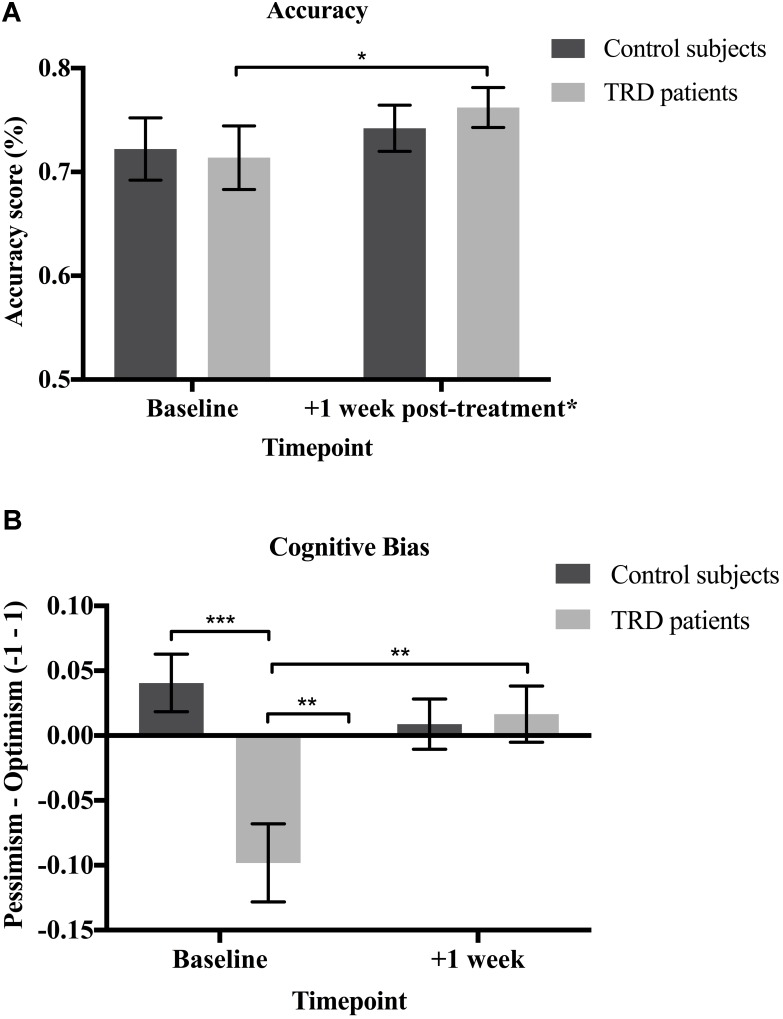
**(A)** Accuracy. Patients were significantly more accurate in their predictions at the follow-up relative to baseline [*t*(14) = 1.857, *p* = 0.042; 95% CI (–0.01, 0.12), *g* = 0.6]. There were no significant changes in accuracy for the control subjects [*t*(14) = 0.603, *p* = 0.556; 95% CI (–0.06, 0.09)]. No between-groups differences in accuracy were found at baseline [*t*(28) = –0.196, *p* = 0.423] or the follow-up [*t*(28) = 0.676, *p* = 0.252; 95% CI (–0.04, 0.08)]. **(B)** Cognitive bias. The greater the digression from zero on the y-axis, the greater the bias. Patients had a significant pessimism bias at baseline [*t*(14) = –3.260, *p* = 0.006; 95% CI (–0.16, –0.03), *g* = 1.1]. No significant biases were found for the controls [*t*(14) = 1.823, *p* = 0.090; 95% CI (–0.09, 0.01)]. Baseline bias scores were significantly lower (i.e., greater pessimism) for the patients than the controls [*t*(28) = 3.704, *p* < 0.001; 95% CI (0.06, 0.22), *g* = 1.3]. Patients’ pessimism bias was significantly reduced [*t*(14) = –2.714, *p* = 0.017] and they no longer displayed any biases following psilocybin treatment [*t*(14) = 0.768, *p* = 0.455; 95% CI (–0.06, 0.02)]. No significant biases were found for the controls [*t*(14) = 0.460, *p* = 0.653; 95% CI (–0.05, 0.03)] and there was no significant between-groups difference [*t*(28) = –0.265, *p* = 0.793; 95% CI (–0.07, 0.05)] at the follow-up. Data expressed as mean ± SEM (*p* < 0.05^∗^; *p* < 0.01^∗∗^; *p* < 0.001^∗∗∗^). ^∗^ Only the TRD patients received psilocybin treatment.

Next, we examined whether there were any changes in the accuracy of participants’ forecasts—defined as the magnitude of discrepancy between predictions and actual event occurrence—between baseline and the post-treatment repeat phase. Closely paralleling the above results, patients were significantly more accurate in their predictions post-treatment (*M* = 0.76, *SD* = 0.07) than at pre-treatment baseline [*M* = 0.71, *SD* = 0.12; *t*(14) = 1.857, *p* = 0.042; 95% CI (-0.01, 0.12), *g* = 0.6] (**Figure 3A**).

As predicted, there were no significant changes in accuracy observed in the non-treated healthy control subjects from baseline (*M* = 0.72, *SD* = 0.12) to the repeat phase [*M* = 0.74, *SD* = 0.09; *t*(14) = 0.603, *p* = 0.556; 95% CI (-0.06, 0.09)]. There were also no significant between-groups differences found at baseline [*t*(28) = -0.196, *p* = 0.423; 95% CI (-0.09, 0.08)] or the follow-up [*t*(28) = 0.676, *p* = 0.252; 95% CI (-0.04, 0.08)] (**Figure 3A**).

### Assessing the Presence of a Pessimism Bias in the TRD Patients

Before proceeding to testing specific hypotheses, some description of the computation of bias is warranted. In order to compute cognitive bias, the life events that actually occurred need to be taken into account. Therefore, different base rates for desirable versus undesirable events do not impede the analysis of bias. Bias scores can range from -1 to 1, with a score of 0 indicating that there is neither an optimism nor pessimism bias. The greater the deviation from 0, the greater the pessimism (negative scores) or optimism (positive scores) bias.

The mean amount of bias shown by the patients at baseline (*M* = -0.10, *SD* = 0.12) was significantly lower than zero; thus confirming a suspected pessimism bias [*t*(14) = -3.260, *p* = 0.006; 95% CI (-0.16, -0.03), *g* = 1.1]. This should be interpreted as consistent with what one would suspect in a study of people with mostly severe depression. Following psilocybin treatment, this pessimism bias was significantly reduced [*M* = 0.02, *SD* = 0.08; *t*(14) = -2.714, *p* = 0.017; 95% CI (-0.21, -0.02), *g* = 0.7] to such an extent that the mean score was no longer significantly different from zero [*t*(14) = 0.768, *p* = 0.455; 95% CI (-0.06, 0.02)] – indicating an effective absence of bias. These results demonstrate that the patients were indeed “unjustifiably” or erroneously pessimistic at baseline and that this cognitive bias was remediated following psilocybin treatment (**Figure 3B**).

The mean amount of bias for the control subjects was negligible and did not significantly differ from zero at baseline [*M* = 0.04, *SD* = 0.09, *t*(14) = 1.823, *p* = 0.090; 95% CI (-0.09, 0.01)] or the repeat phase [*M* = 0.01, *SD* = 0.07, *t*(14) = 0.460, *p* = 0.653; 95% CI (-0.05, 0.03)]. This demonstrates an effective absence of bias (i.e., no appreciable optimism or pessimism bias) in the control subjects at both baseline and the follow-up (**Figure 3B**).

Not surprisingly, bias scores were significantly lower (i.e., greater pessimism) for patients (*M* = -0.10, *SD* = 0.12) than controls at baseline [*M* = 0.04, *SD* = 0.09; *t*(28) = 3.704, *p* < 0.001; 95% CI (0.06, 0.22), *g* = 1.3], but there was no significant difference for the repeat phase [*t*(28) = -0.265, *p* = 0.793; 95% CI (-0.07, 0.05)]. These results demonstrate that the patients were more pessimistic than the controls at baseline, but this bias was alleviated to control levels following psilocybin treatment (**Figure 3B**).

### Relationship Between Cognitive Biases and Depressive Symptoms

**FIGURE 4:**
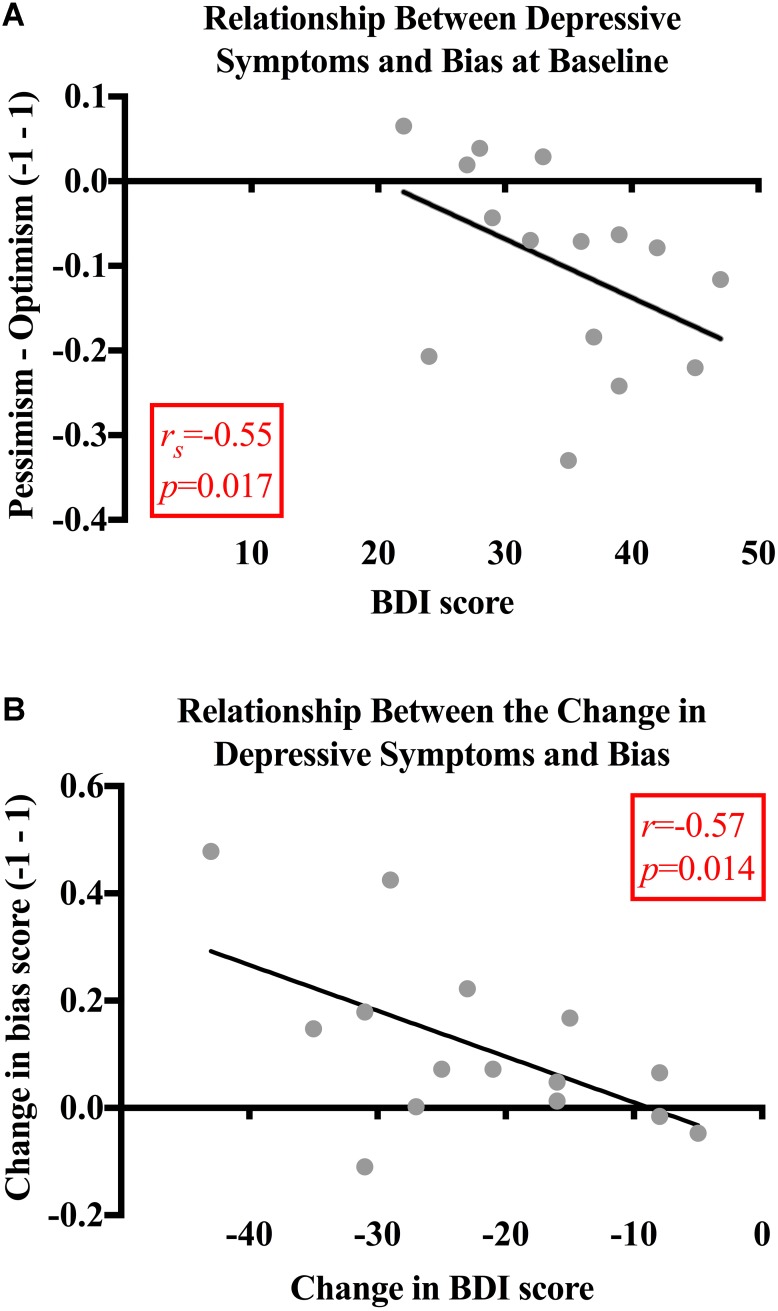
**(A)** Relationship between depressive symptoms and bias. At baseline, the patients’ bias scores were moderately and significantly related to their BDI scores (*r_s_* = -0.55, *p* = 0.017). No significant relationships were found between BDI and bias scores for the control subjects (*r_s_* = -0.22, *p* = 0.219). **(B)** Relationship between the change in depressive symptoms and bias. Bias and BDI scores changed significantly more for the patients than the control subjects’ bias [*t*(28) = -3.167, *p* = 0.004; 95% CI (-0.24, -0.05), *g* = 1.2] and BDI scores [*t*(28) = 7.228, *p* < 0.001; 95% CI (15.09, 27.04), *g* = 2.6]. The decrease in BDI score was significantly related to the decrease in pessimism for the patients (*r* = -0.57, *p* = 0.014). There was no significant relationship found between the change in BDI and bias scores (*r_s_* = -0.007, *p* = 0.980) for the control subjects.

Next, we investigated any potential relationships between baseline pessimism and depressive symptoms. Before psilocybin, the patients’ bias scores were significantly related to their BDI scores (*r_s_* = -0.55, *p* = 0.017). This finding demonstrates that greater pessimism (i.e., lower bias scores) was associated with more severe depressive symptoms in the patients. No significant relationships were found between BDI and bias scores for the control subjects (*r_s_* = -0.22, *p* = 0.219) (**Figure 4A**).

When analyzing the magnitude of change in scores between baseline and the follow-up, it was found that the bias (*M* = 0.11, *SD* = 0.16) and BDI scores (*M* = -22.20, *SD* = 10.88) changed significantly more for the patients than the control subjects [bias: *M* = -0.03, *SD* = 0.73, *t*(28) = -3.167, *p* = 0.004, 95% CI (-0.24, -0.05), *g* = 1.2; BDI: *M* = -1.13, *SD* = 2.99, *t*(28) = 7.228, *p* < 0.001, 95% CI (15.09, 27.04), *g* = 2.6]. The decrease in BDI score was significantly related to the decrease in pessimism for the patients (*r* = -0.57, *p* = 0.014; **Figure 4B**). There was no significant relationship found between the change in BDI and bias scores (*r_s_* = -0.007, *p* = 0.980) for the control subjects.

## Discussion

The present study sought to investigate the effects of psilocybin on pessimism bias in patients with TRD. Before treatment with psilocybin, patients were excessively and unrealistically pessimistic when predicting the occurrence of future life events – and this pessimism was significantly correlated with the severity of their depressive symptoms. One week after treatment, the patients’ pessimism was alleviated and their depressive symptoms greatly improved; moreover, the magnitude of change in both variables was related – such that as their depression improved, so did their ability to accurately forecast their future. No such bias nor change in forecasting was seen in a matched control group assessed over an equivalent time period. Taken together, these findings indicate that the psychologically supportive administration of psilocybin remediates negative cognitive biases characteristic of severe depression – enabling individuals to forecast their futures more accurately, unfettered by unrealistic pessimism.

### Depressive Symptoms, Biases and Accuracy of Predictions

The alleviation of depressive symptoms one week after psilocybin treatment found here is consistent with previous research demonstrating the therapeutic potential of psychedelic compounds (Carhart-Harris and Goodwin, 2017). The ability of psychedelics to disrupt reinforced patterns of negative thought and behavior by disintegrating the patterns of brain activity upon which they rest may account for their positive therapeutic effects (Carhart-Harris et al., 2014; Watts et al., 2017). A single dose of the natural psychedelic brew prepared from Amazonian plants, ayahuasca, had rapid, and sustained antidepressant effects in patients with recurrent MDD (Osório Fde et al., 2015). Moreover, a single dose of psilocybin has also been shown to induce long-lasting reductions in anxiety and depression as well as increases in quality of life, life meaning, and optimism in patients suffering from depression and anxiety reactive to advanced-stage cancer (Grob et al., 2011; Griffiths et al., 2016; Ross et al., 2016). Specifically regarding TRD, we previously showed significant reductions in depressive symptoms 1-week after psilocybin treatment, with maximal reductions seen at 5 weeks post-treatment. For many of the patients, these reductions persisted 6 months later. Enduring improvements in psychological wellbeing (Griffiths et al., 2008), trait openness and optimism (MacLean et al., 2011; Carhart-Harris et al., 2016b) have also been observed in healthy volunteers following a single dose of a psychedelic drug.

As predicted by the cognitive-bias model of depression (Beck, 1967, 1976), here we found a significant pessimism bias in the TRD patients when predicting the likelihood of future events prior to psilocybin treatment. Consistent with the depressive bias hypothesis—i.e. the theory that as depressive symptoms increase in severity, judgments become more negatively biased (Beck, 1976)—we found greater pessimism among patients with more severe depressive symptoms. This is in line with previous studies demonstrating a relationship between depression and pessimism (Peterson and Seligman, 1984; Hollon et al., 1986; Beck et al., 1988; Hill et al., 1989). It also opens up the possibility of using pessimism in healthy samples as a proxy of depression in clinically depressed samples. The finding here that patients were unrealistically pessimistic about the likelihood of positive life events befalling them prior to psilocybin treatment appeared not to be entirely justified by poor life circumstances, as the patients’ predictions (e.g., of the likelihood of desirable life events) underestimated their actual rate of occurrence. Moreover, the rates at which desirable and undesirable events actually occurred did not differ from the control subjects, who lacked such biases. This is consistent with classic depression research demonstrating that depressed patients present with excessively negative perspectives that are disproportionate to their true life circumstances (Beck, 1967; Strunk et al., 2006; Strunk and Adler, 2009).

One week after psilocybin treatment, we found that the patients’ pessimism was alleviated and they no longer showed any cognitive biases. In line with this finding, previous research has demonstrated sustained increases in optimism following psilocybin treatment in patients suffering from depression and anxiety reactive to advanced-stage cancer (Grob et al., 2011; Griffiths et al., 2016; Ross et al., 2016). Long-lasting improvements in optimism have also been observed in healthy volunteers following a single dose of a psychedelic (MacLean et al., 2011; Carhart-Harris et al., 2016b) and there is also evidence that recreational psychedelic users are more optimistic (or less pessimistic) than non-users (Grob et al., 1996). Psychedelic compounds have previously been shown to increase positive mood (Kraehenmann et al., 2015; Schmid et al., 2015) and often induce lasting changes in attitudes and behavior associated with a more positive outlook (Savage, 1962; McGlothlin and Arnold, 1971; Griffiths et al., 2006, 2008; Studerus et al., 2011). Here, we found that patients predicted significantly more desirable events post-treatment; making more accurate predictions that better reflected their actual life situation. No cognitive biases were found among the control subjects.

The lack of cognitive bias in the controls found here is in contrast to previous research suggesting that healthy individuals have a tendency towards an optimism bias (Taylor and Brown, 1988; Weinstein, 1989; Sharot et al., 2007). Evidence suggests that healthy people are worse at incorporating information about a more negative than expected future, and instead are more likely to update their beliefs in response to better-than-expected information, resulting in an unrealistic optimism (Izuma and Adolphs, 2011; Sharot et al., 2011; Garrett et al., 2014; Korn et al., 2014; Garrett and Sharot, 2017). It has been shown that this unrealistic optimism is maintained due to a selective update failure and the diminished neural coding of undesirable information (Sharot et al., 2011). Dismissing and/or denying undesirable information may serve an adaptive function by enhancing exploratory behavior and reducing stress and anxiety (Scheier et al., 1989; Taylor et al., 2000; Varki, 2009). This is in line with research showing a relationship between mild depression and accuracy in predictions, with more severe depression being associated with clear pessimism (Strunk et al., 2006). However, an unrealistic optimism has drawbacks as well, e.g., in terms of underestimating risks and so failing to adopt preventative and/or protective measures (Shepperd et al., 2013).

This idea that optimism is conducive to positive mental health has been critiqued recently however, e.g., with arguments made that improved methodologies are required to elucidate such biases and an accurate perception of reality is most important for mental health (Colvin and Block, 1994; Shah et al., 2016) – see also Letheby (2016) in specific relation to the psychedelic experience. Entertaining worst-case scenarios when anticipating future events may help one’s ability to cope with negative outcomes, through contingency plans and preparedness. In this sense, there are benefits of having a pessimistic attitude and expecting an undesirable outcome when facing events of unknown emotional impact (Nesse, 2000), such as not engaging in risky behavior (Gibson and Sanbonmatsu, 2004) and avoiding disappointment by setting low expectations (Norem and Cantor, 1986; Shepperd and McNulty, 2002). However, an unrealistic pessimism about the future also represents a key cognitive feature in MDD (Pyszczynski et al., 1987; Lavender and Watkins, 2004), as expressed in the cognitive triad (Beck, 1967). It follows that a balanced, reality-focused perspective is optimal for mental health – as unrealistic optimism may encourage negligent and reckless behavior that could result in catastrophe, and unrealistic pessimism may lead to avoidant behavior, passivity, low mood, vulnerability to MDD and perhaps related disorders (Hecht, 2013).

The neurobiology of optimism versus pessimism is poorly understood. It is well known however, that serotonergic mechanisms are involved in mood states and cognitive styles (Meneses, 1999; Young and Leyton, 2002; Cowen and Sherwood, 2013); for example, the 5-HT_2A_ receptor, which is the key receptor through which psychedelics elicit their signature effects (Glennon et al., 1984; Nichols, 2017), is upregulated during states of low synaptic 5-HT (Cahir et al., 2007) as well as in unmedicated depressed patients (Bhagwagar et al., 2006) and individuals scoring high on neuroticism (Frokjaer et al., 2008, 2010) and dysfunctional attitudes (Meyer et al., 2003) – an analog of trait pessimism. We recently proposed a mechanistic model by which psychedelic-induced 5-HT_2A_R signaling rapidly induces an acute state of plasticity in which an enriched context may lead to the revision of cognitive biases (Carhart-Harris and Goodwin, 2017; Carhart-Harris and Nutt, 2017; Carhart-Harris et al., 2018). MDD is associated with structural brain changes that clinically reflect the presence of cognitive and emotional biases (Serafini et al., 2014). Psychedelics have been shown to temporarily deconstruct the default-mode network (DMN) (Carhart-Harris et al., 2012; Palhano-Fontes et al., 2015; Speth et al., 2016), a network in the brain associated with ruminative thought (Berman et al., 2011), self-reflection (Johnson et al., 2002) and introspection more generally (Fleming et al., 2010; Qin and Northoff, 2011) – although the DMN appears to recover its integrity as the effects of a psychedelic wear off, with a potential increase in integration post treatment with psilocybin for TRD (Carhart-Harris et al., 2017b; Watts et al., 2017). To account for the post-treatment brain effects of psilocybin, we recently proposed a “reset” mechanism by which acute modular disintegration may enable a subsequent reintegration and resumption of normal functioning, accompanied by improvements in mood (Carhart-Harris et al., 2017b). We hope to explore the relationship between DMN properties before, during and after psilocybin in future studies and assess its relationship to optimism/pessimism. A functional imaging paradigm that incorporates the actual forecasting of future life events (prospection) might be interesting to explore in this context.

### Methodological Strengths

The results reported here substantially extend findings of previous research demonstrating increased optimism following psychedelic drug use (Barbosa et al., 2009; Griffiths et al., 2006, 2011; Carhart-Harris et al., 2016b). Previous studies of psychedelics-induced optimism have used subjective measures, whereas the present study utilized a behavioral instrument to more objectively measure optimism/pessimism. By analyzing the effects of psilocybin on the prediction of one’s personal future, this study addresses an important facet of the cognitive triad, i.e., a negative attitude towards oneself, the environment and the future. To our knowledge, this is the first study to analyze the effects of a psychedelic compound on cognitive biases in patients with TRD and also the first to address psychedelic-induced changes in biases that are not purely subjective and self-referential, but instead feature a behavioral aspect, requiring the predictions of future life events – the accuracy of which can later be assessed based on what is reported to have actually occurred in the individual’s life.

### Limitations

It is necessary to address some limitations of this study. It formed part of an open-label clinical trial (Carhart-Harris et al., 2016a) with a small sample size, and although a control group was recruited to examine test re-test reliability on these measures, the controls were healthy subjects not exposed to the same treatment procedures. The cross-sectional design of this study and the inability to confirm the present data using further prospective follow-ups must also be considered. It is possible that a natural decline of depressive symptoms contributed to our findings. Additionally, since treatment with psilocybin involved more than just drug administration (e.g., psychological support before and after the psilocybin dosing sessions), it is possible that some drug-unrelated factor(s) contributed to the changes in cognitive bias observed here. It should be emphasized, however, that such non-drug components are considered an integral part of the therapeutic model and cannot easily be extricated from it without collapsing the model itself (e.g., see Carhart-Harris et al., 2018). The specificity of our main results also requires careful consideration. It is unclear whether the reported changes in cognitive bias found here following psilocybin treatment were selective for these outcomes or rather an epiphenomenon of the treatment’s core effects on depressive symptoms. It might be that cognitive biases are an inextricable part of depression and so an impact on one of these factors will necessarily affect the other. The question of causality is of central relevance here, and only further research can elucidate this. In this context, we would like to propose that there is a common mediating factor at play, driving both the improvements in mental health and changes in biases seen here – as well as elsewhere with psilocybin and other psychedelics (Krebs and Johansen, 2013; Hendricks et al., 2015; Carhart-Harris et al., 2016b; Watts et al., 2017).

## Conclusion

In conclusion, this study sought to investigate the effects of psilocybin with psychological support using the Prediction of Future Life Events task in patients with TRD. To our knowledge, this is the first study using a behavioral measure to objectively address cognitive biases integral to depression and how these change post-treatment with a psychedelic. The present findings suggest that the psilocybin with psychological support treatment model may alleviate pessimism bias in depression, giving patients a clearer, more accurate outlook on their future. Further controlled studies are warranted to better determine the causality, reliability, specificity and durability of these findings.

## Author Contributions

RC-H conceptualized the study design. TL and RC-H collected the data and TL plotted, analyzed and interpreted the data. TL wrote the manuscript and RC-H provided edits.

## Conflict of Interest Statement

The authors declare that the research was conducted in the absence of any commercial or financial relationships that could be construed as a potential conflict of interest.
