# Supplementary material for: More Realistic Forecasting of Future Life Events After Psilocybin for Treatment-Resistant Depression
Source: Front Psychol. 2018 Oct 12;9:1721. doi: 10.3389/fpsyg.2018.01721 (PMC6194345; doi:10.3389/fpsyg.2018.01721)
Supplement: Supplementary file 1 [file Table_1.DOCX]

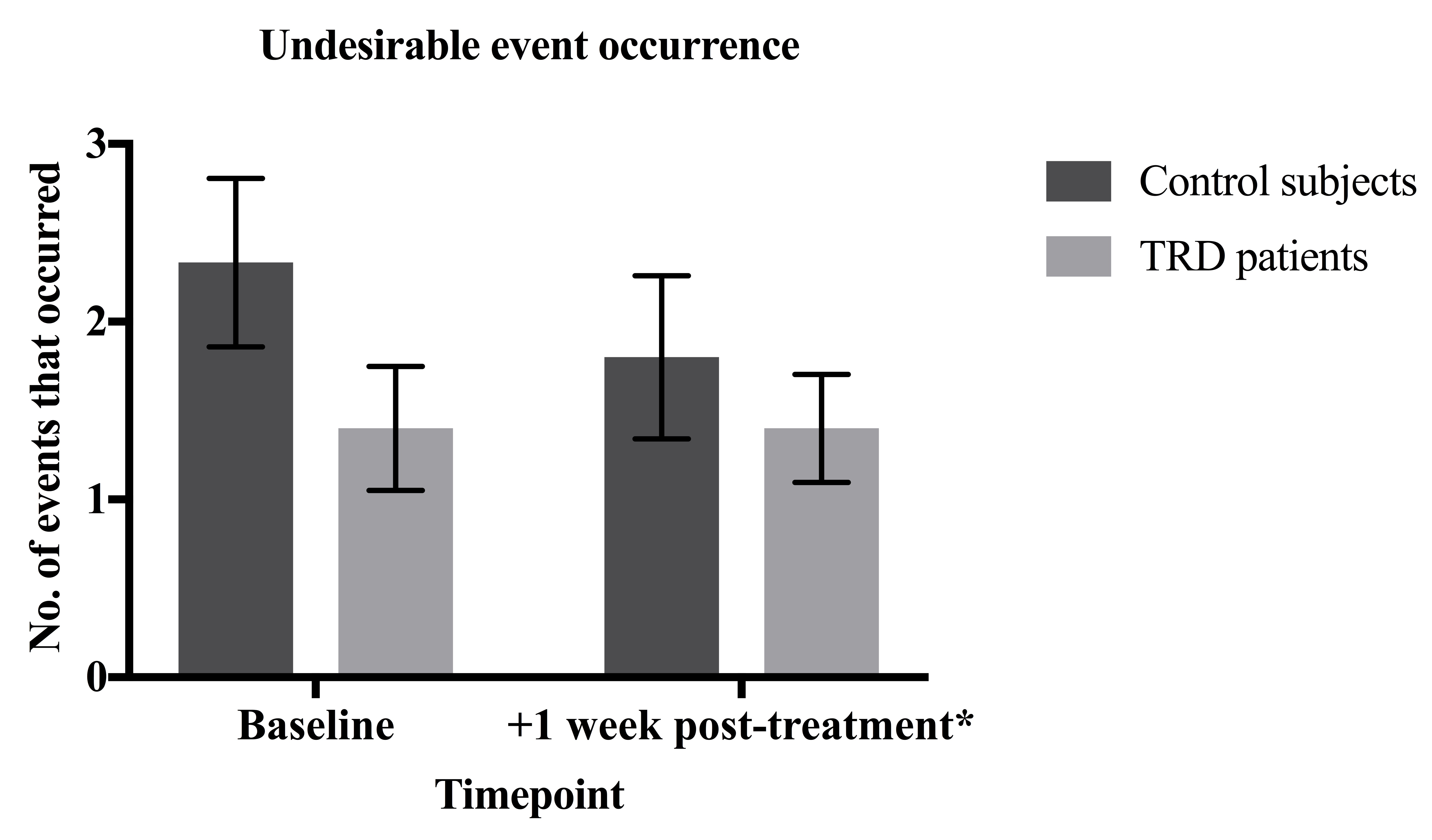

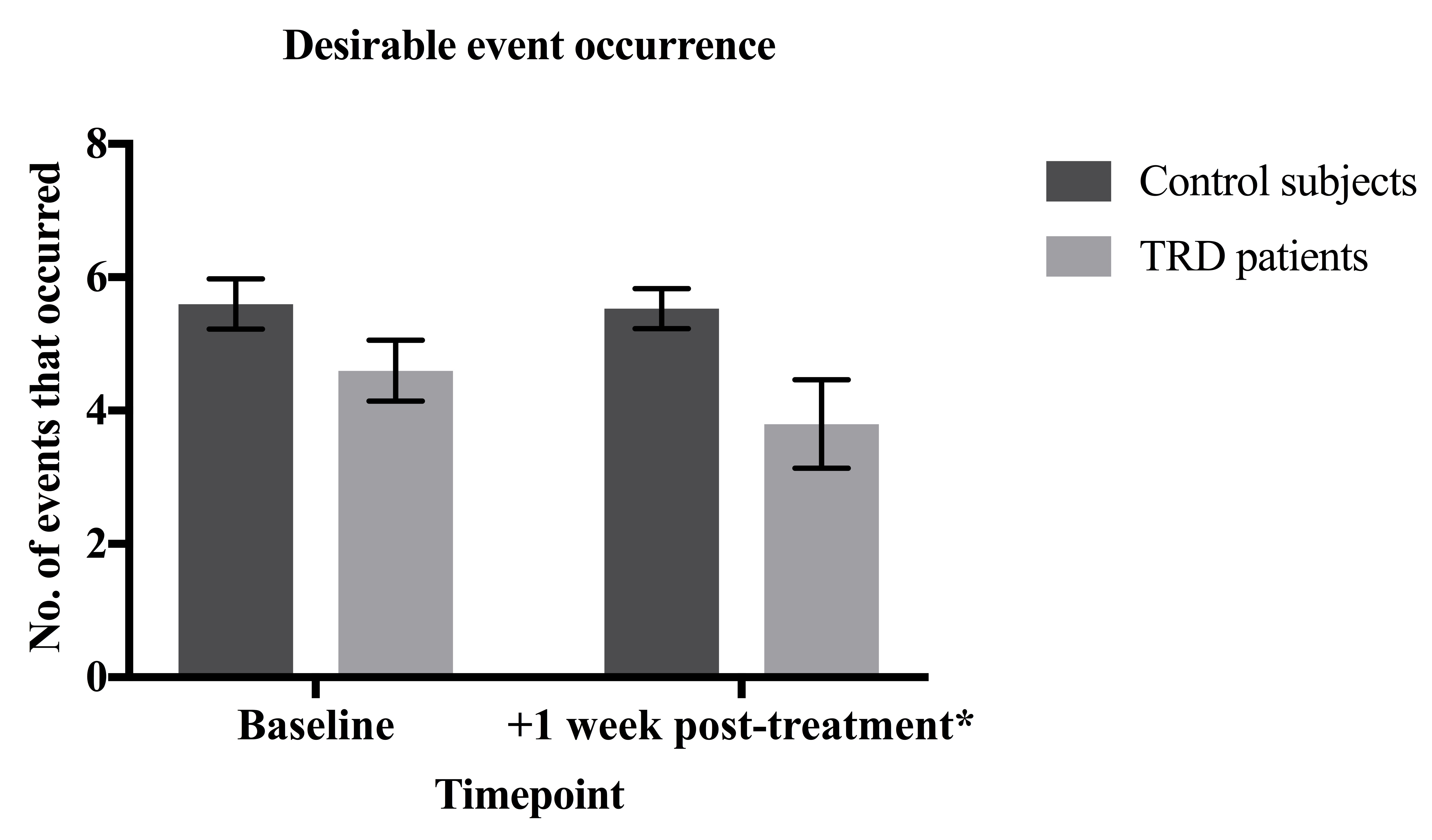

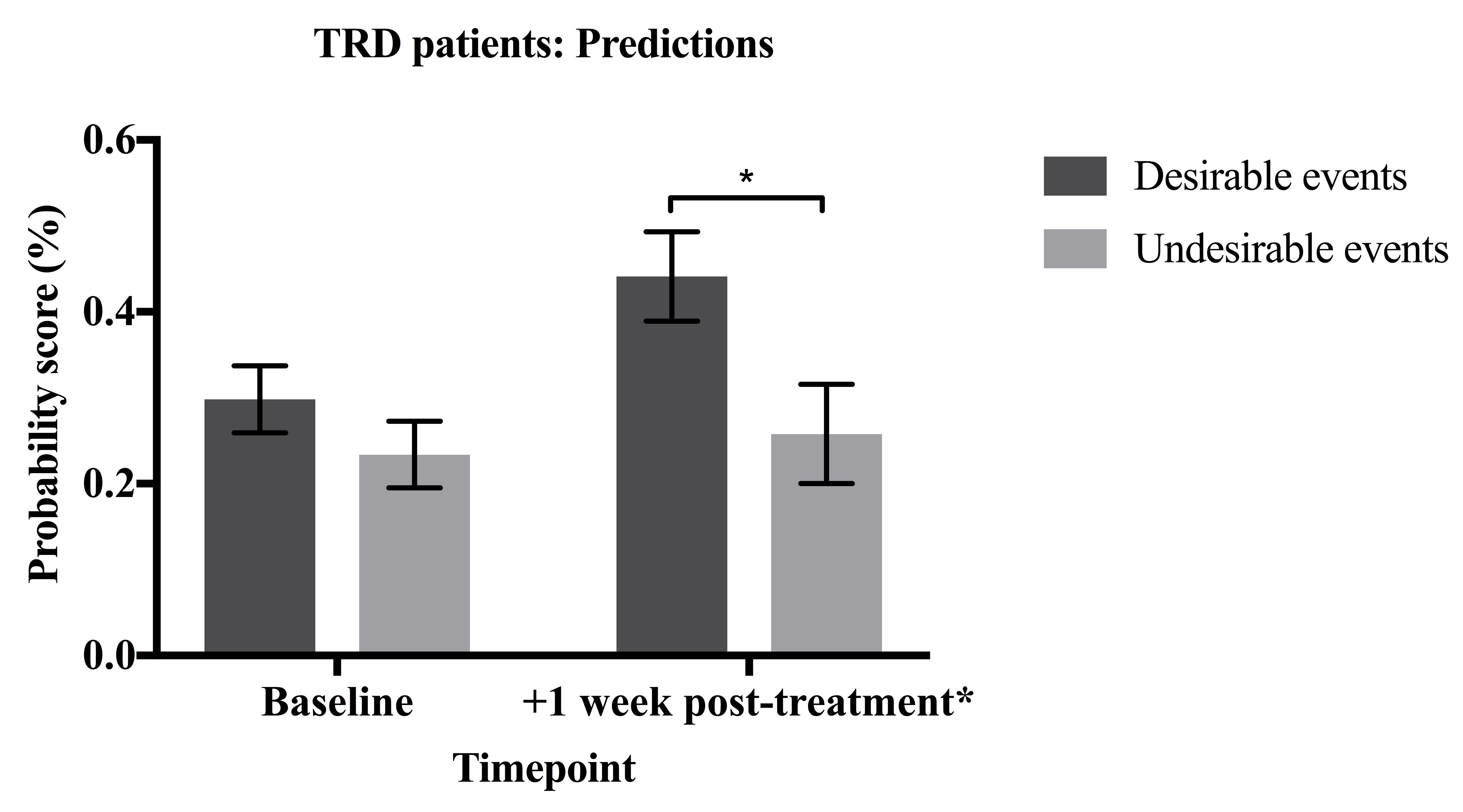

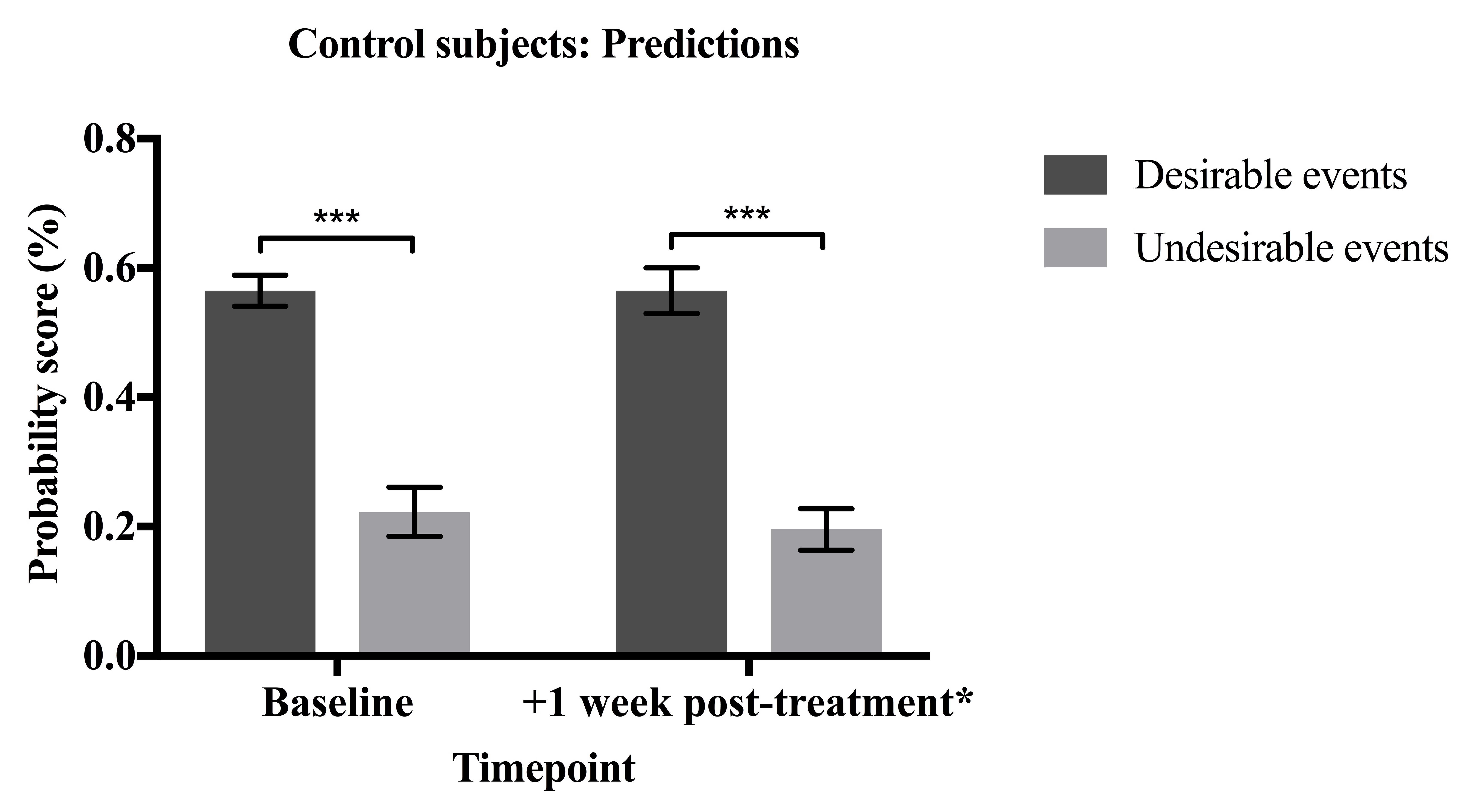


**(A)**

**(B)**

**(C)**

**(D)**

**Figure S1. (A) Control subjects: Predictions.** This chart shows the control subjects’ predicted probability of event occurrence. Control subjects gave higher probability estimates for desirable (*M*=0.57, *SD*=0.09) than undesirable life events (*M*=0.22, *SD*=0.16; *t*(14)=8.492, *p*<0.001) at baseline. The control subjects also gave higher probability estimates for desirable (*M*=0.57, *SD*=0.14) than undesirable (*M*=0.20, *SD*=0.12) life events at the follow-up (*t*(14)=8.399, *p*<0.001), and did not differ from baseline in their second predictions of desirable (*t*(14)=-0.0001, *p*=1.000) or undesirable events (*t*(14)=0.686 *p*=0.504). **(B) TRD patients: Predictions.** This chart shows the TRD patients’ predicted probability of event occurrence. When predicting life events at baseline, the patients gave similar probability estimates for desirable (*M*=0.29, *SD*=0.15) and undesirable life events (*M*=0.23, *SD*=0.15, *t*(14)=1.037, *p*=0.317). In contrast to their pre-treatment predictions, post-treatment, patients gave significantly higher probability estimates for desirable (*M*=0.44, *SD*=0.20) than undesirable life events (*M*=0.25, *SD*=0.22, *t*(14)=2.322, *p*=0.036). **(C) Desirable event occurrence.** This chart shows the rate at which the desirable events actually occurred within the ensuing 30 days after the participants’ predictions. There were no significant between-groups differences found in the rates at which desirable (patients: *M*=4.60, *SD*=1.76; controls: *M*=5.60, *SD*=1.45; *U*=75.000, *p*=0.113) events occurred within the 30-day period from baseline. This was also the case at the follow-up as there were no significant between-groups differences found in the rates at which desirable (patients: *M*=3.80, *SD*=2.57; controls: *M*=5.53, *SD*=1.19; *U*=70.000, *p*=0.072) events actually occurred within the second 30-day period. **(D) Undesirable event occurrence.** This chart shows the rate at which the undesirable events actually occurred within the ensuing 30 days after the participants’ predictions. There were no significant between-groups differences found in the rates at which undesirable (patients: *M*=1.40, *SD*=1.35 controls: *M*=2.33, *SD*=1.84; *U*=78.500, *p*=0.148) events occurred within the 30-day period from baseline. This was also the case at the follow-up as there were no significant between-groups differences found in the rates at which undesirable (patients: *M*=1.40, *SD*=1.18; controls: *M*=1.80, *SD*=1.78; *U*=105.000, *p*=0.741) events actually occurred within the second 30-day period. Data expressed as mean ± SEM [*p*<0.05*; *p*<0.001***].

* Only the TRD patients received psilocybin treatment.
